# The endophyte secondary metabolite phenazine-1-carboxylic acid promotes tanshinone biosynthesis in *Salvia miltiorrhiza* callus

**DOI:** 10.1007/s11418-026-02075-w

**Published:** 2026-10-05

**Authors:** Miho Hamai, Anju Ishii, Nozomi Sakamoto, Hiroki Fujioka, Junko Tsukioka, Tomoe Ohta, Seikou Nakamura, Takahiro Matsumoto

**Affiliations:** Kyoto Pharmaceutical University, Misasagi, Yamashina-ku, Kyoto, 607-8412 Japan

**Keywords:** *Salvia miltiorrhiza*, Tanshinone, Endophytes, Secondary metabolism, Biosynthesis of tanshinones

## Abstract

*Salvia miltiorrhiza* is a traditional medicinal plant widely used to treat cardiovascular and cerebrovascular diseases. Tanshinones, including cryptotanshinone, a major bioactive constituent, exhibit various pharmacological activities. Therefore, methods for efficiently increasing tanshinone production are urgently needed. Recently, plant endophytes have attracted considerable attention as novel elicitors that promote the production of valuable bioactive compounds. In the present study, we investigated the effects of secondary metabolites produced by endophytes on the biosynthesis and accumulation of tanshinones in *S. miltiorrhiza. Pseudomonas chlororaphis* KPUPH-SMC was identified as an endophyte capable of promoting tanshinone production, and phenazine-1-carboxylic acid (PCA) was identified as the metabolite responsible for this activity. Our transcriptome analysis further demonstrated that PCA significantly upregulated the gene expression of tanshinone biosynthetic enzymes (GGPS, CPS, and KSL1). These findings suggest that endophyte-derived secondary metabolites can be used to enhance the biosynthesis of valuable compounds in medicinal plants. This approach represents a promising strategy to improve the quality of herbal medicines.

**Graphical abstract:**

**Figure:**
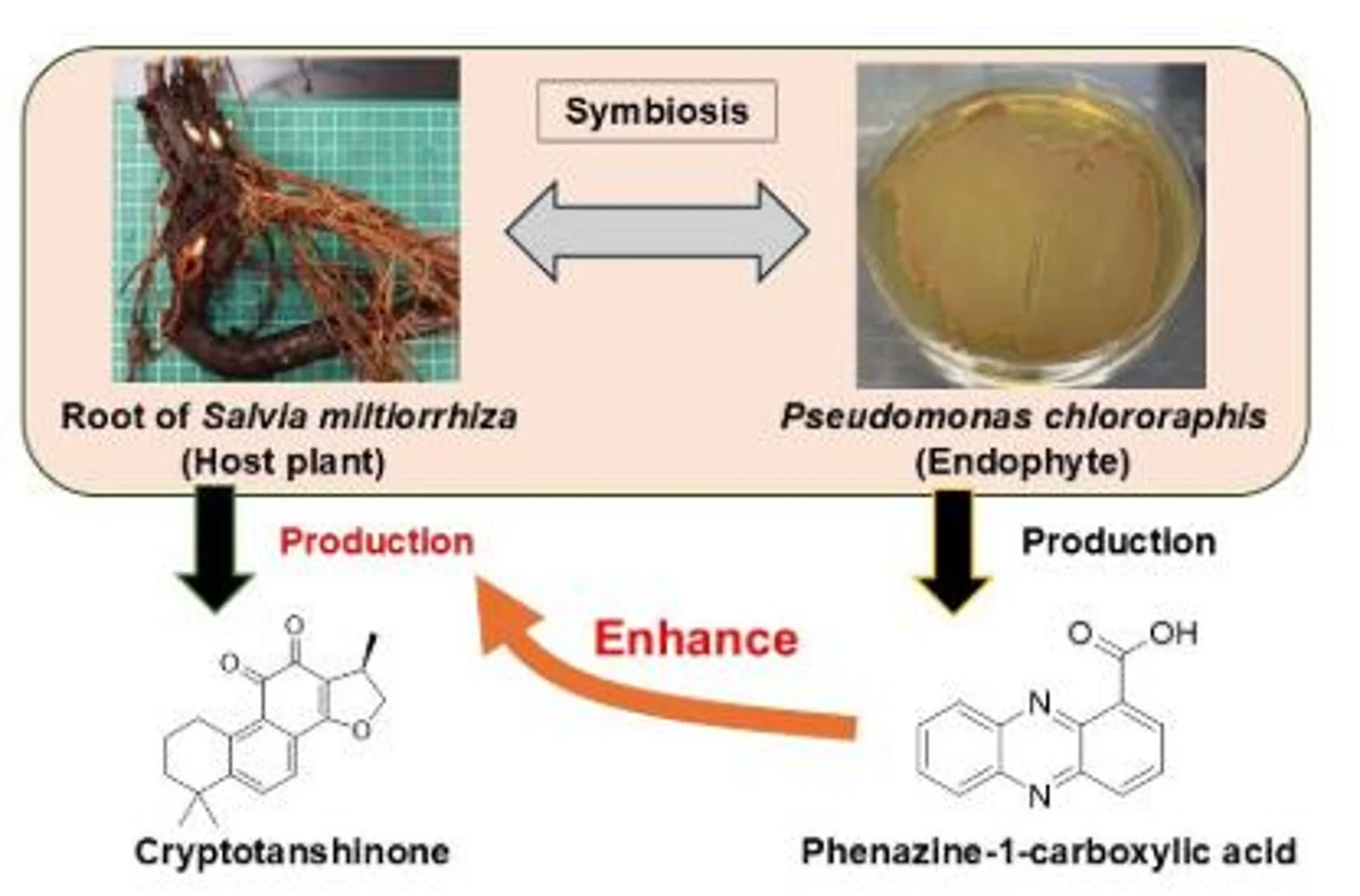

**Supplementary Information:**

The online version contains supplementary material available at https://doi.org/10.1007/s11418-026-02075-w.

## Introduction

**Fig. 1:**
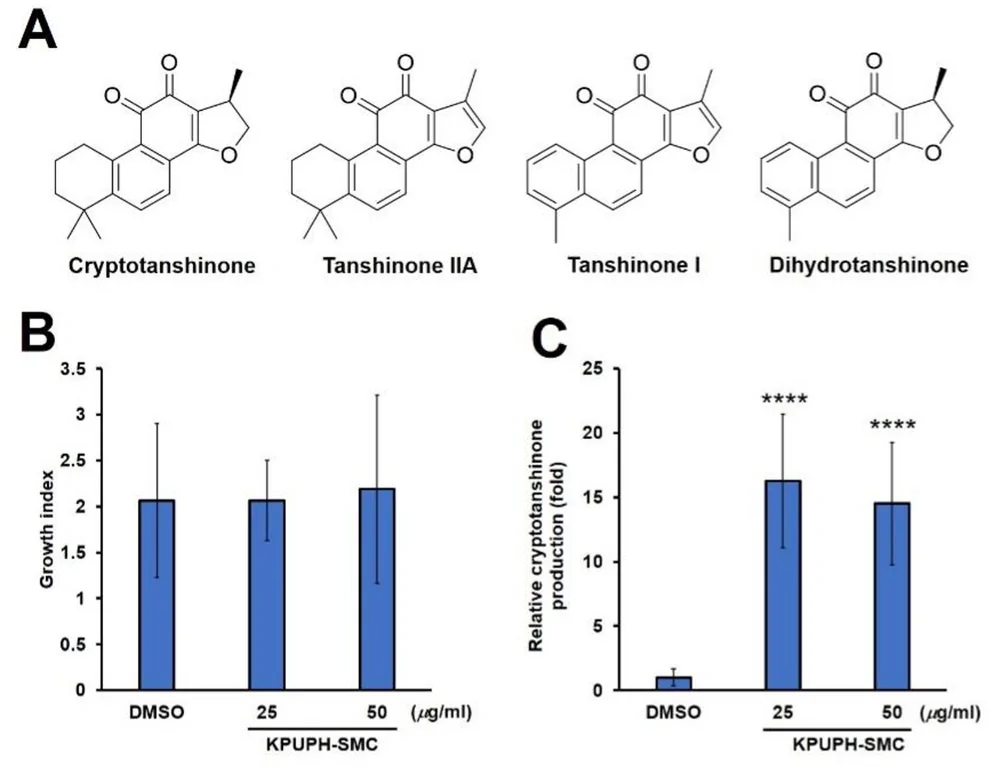
Tanshinones in *S. miltiorrhiza* and effects of KPUPH-SMC EtOAc extract on cryptotanshinone production in *S. miltiorrhiza* callus. **A** Chemical structures of tanshinones; **B** Growth index of callus treated with DMSO (control) or KPUPH-SMC EtOAc extract (25 and 50 *µ*g/ml) for two weeks; **C** Relative cryptotanshinone production in callus under the same treatment conditions, as determined by LC-MS/MS analysis. Data are presented as means ± SD, *n* = 6, *P < 0.001 versus the control culture

*Salvia miltiorrhiza* (*S. miltiorrhiza*) Bunge is a perennial herb of the Lamiaceae family that is widely used as a medicinal plant in traditional Chinese medicine [1]. Its dried roots contain abundant bioactive compounds, including lipophilic tanshinones (e.g., cryptotanshinone, tanshinone ⅡA, tanshinone Ⅰ, and dihydrotanshinone) (Fig. 1A) [2] and hydrophilic phenolic acids (e.g., salvianolic acids) [3]. These compounds are well-known for their preventive and therapeutic efficacy against cardiovascular and hematological diseases [4]. Recent research has highlighted their diverse pharmacological activities, including their potential for inhibiting cancer, thereby expanding their medical significance [5]. Therefore, stabilizing and increasing the content of these key medicinal components is important for improving the quality of *S. miltiorrhiza* [6].

Medicinal plants can establish symbiotic relationships with diverse microorganisms, and these endophytes have been reported to promote the accumulation of bioactive compounds in host plants [7, 8]. For *S. miltiorrhiza*, several endophytes were reported to promote the secondary metabolites accumulation in the host plant. For example, water extract of mycelia of endophytic fungus *Chaetomium globosum* D38 from the roots of *S. miltiorrhiza* increases the accumulation of tanshinones of the host plant [9]. Next, polysaccharide fraction from the endophytic fungus *Trichoderma atroviride* D16 significantly increases the transcriptional levels of genes related to the tanshinone biosynthesis pathway [10]. These findings suggest that primary metabolites produced by endophytes affect the regulation of tanshinone biosynthesis in *S. miltiorrhiza*. On the other hand, endophytes including fungi and bacteria produce chemically diverse bioactive secondary metabolites [11], therefore, we discussed that secondary metabolites produced by endophytes may affect the regulation of tanshinone biosynthesis in *S. miltiorrhiza*.

In this study, we used *S. miltiorrhiza* callus as a model system because plant callus cultures provide a homogeneous and experimentally tractable system in which environmental conditions and elicitor treatments can be precisely controlled, making them widely used to investigate the regulation of secondary metabolite biosynthesis [12, 13]. We then investigated the effects of endophyte-derived secondary metabolites on tanshinone biosynthesis. Transcriptome analysis was performed to comprehensively examine changes in gene expression induced by treatment with these metabolites. Furthermore, we aimed to elucidate the regulatory mechanisms underlying tanshinone biosynthesis.

## Results and discussion

The endophyte, KPUPH-SMC was isolated from the roots of *S. miltiorrhiza* and cultured on potato dextrose agar. Based on previous reports demonstrating that endophytic fungi can enhance the production of bioactive compounds in medicinal plants, we initially focused on isolating the endophytic fungi from *S. miltiorrhiza*. However, during the isolation process, strain KPUPH-SMC was obtained and subsequently identified as a bacterial endophyte. In this study, the 16 S rRNA gene of KPUPH-SMC was selected and sequenced using nuclear ribosomal DNA as a template. The results of the BLAST searches for the KPUPH-SMC strain showed that the 16 S rRNA gene sequences of this isolate shared high homology with those of *Pseudomonas chlororaphis*. Based on the molecular data, strain KPUPH-SMC was identified as *P. chlororaphis.* The purpose of the present study is to find the secondary metabolites of microorganisms that affect the accumulation of tanshinones biosynthesis in the *S. miltiorrhiza.* Therefore, to avoid extracting polar media components, primary metabolites, and sugars, we prepared extract of *P. chlororaphis* KPUPH-SMC using ethyl acetate (EtOAc) as a moderate polarity solvent [14–16].

The EtOAc-soluble fraction was obtained from agar that had been cultured at 28 °C for two weeks and then milled. The effects of KPUPH-SMC EtOAc extract (25 and 50 *µ*g/ml), dissolved in dimethyl sulfoxide (DMSO), on callus growth were evaluated by measuring the fresh weight of callus before cultivation and after two weeks of culture, followed by calculating the growth index. The results showed that treatment with the KPUPH-SMC EtOAc extract did not significantly affect cell growth compared with the control (Fig. 1B). The callus was lyophilized, ultrasonically extracted with isopropanol, and analyzed using LC-MS/MS to evaluate quantity of cryptotanshinone. LC-MS/MS analysis of cryptotanshinone was performed using an LC-MS/MS system as reported previously with modifications [17]. Cryptotanshinone production was significantly higher in the extract-treated samples than in the controls (Fig. 1C). In addition, we couldn’t detect cryptotanshinone, tanshinone I, tanshinone IIA, and dihydrotanshinone in KPUPH-SMC EtOAc extract using LC-MS/MS analysis [17–19] (Fig. S1). These results suggest that secondary metabolites produced by the endophyte function as elicitors that activate secondary metabolism, leading to enhanced cryptotanshinone accumulation in *S. miltiorrhiza* callus without inhibiting cell growth.

**Fig. 2:**
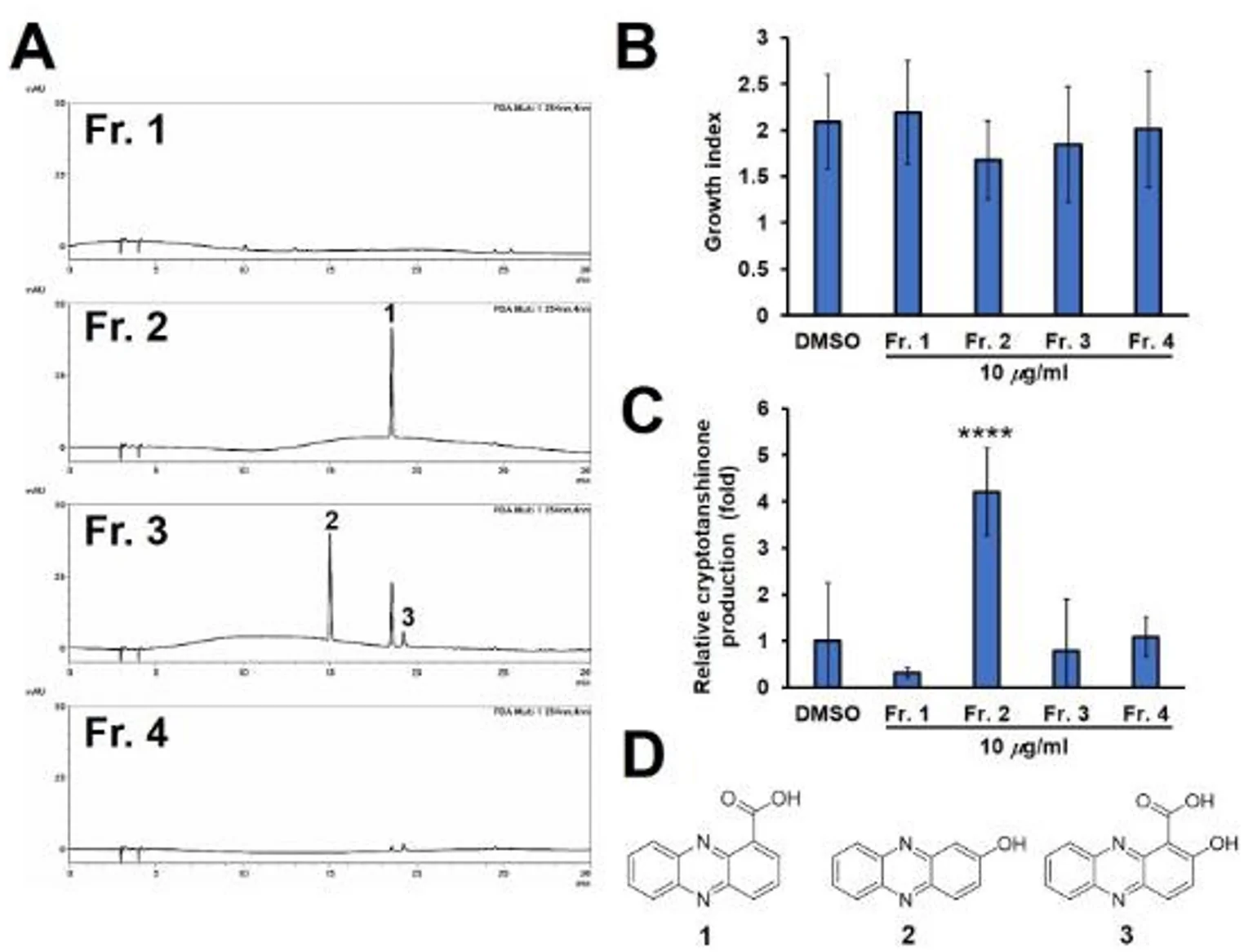
Identification of active compounds in the KPUPH-SMC EtOAc extract that increase cryptotanshinone production. **A** UV-HPLC chromatograms (λ = 254 nm) of four fractions (Fr. 1–4) obtained by reversed-phase silica gel column chromatography of the EtOAc extract; **B** Growth index of *S. miltiorrhiza* callus treated with DMSO (control) or each fraction (Fr. 1–4) at 10 *µ*g/ml for two weeks; **C** Relative cryptotanshinone production in callus treated with each fraction under the same conditions, as determined by LC-MS/MS analysis; **D** Chemical structures of the compounds isolated from Fr. 2 and Fr. 3, identified as phenazine-1-carboxylic acid (**1**), 2-hydroxyphenazine (**2**), and phenazine-1-carboxylic acid 2-ol (**3**). Data are presented as means ± SD, *n* = 6, *P < 0.001 versus the control culture

Next, to identify the active compounds in the KPUPH-SMC extract of the endophyte that increases cryptotanshinone production in the callus, the EtOAc-soluble fraction was fractionated by reversed-phase silica gel column chromatography to yield four fractions (Fr.1–4), which were subsequently analyzed by UV-HPLC at 254 nm (Fig. 2A). Murashige and Skoog medium samples supplemented with DMSO or KPUPH-SMC fractions (Fr.1–4) re-dissolved in DMSO at 10 *µ*g/ml were prepared. Calluses were inoculated into each medium and cultured for two weeks, followed by quantitative analysis using LC-MS/MS to evaluate cryptotanshinone production. LC-MS/MS analysis of cryptotanshinone was performed using an LC-MS/MS system as reported previously with modifications [17]. The results showed that although Fr. 2 slightly reduced the growth index compared with the DMSO control (Fig. 2B), Fr. 2 significantly increased cryptotanshinone production in the callus (Fig. 2C). The UV-HPLC chromatogram of Fr. 2 revealed a major component that, was presumed to play a key role in the observed activity. Therefore, we isolated this compound using HPLC. Furthermore, in addition to the compound identified in Fr. 2, the UV-HPLC chromatogram of Fr. 3 indicated the presence of two additional compounds. These compounds were further isolated and purified using HPLC. The structures of these compounds were characterized by ^1^H and ^13^C NMR analyses and confirmed by comparing their NMR and MS data with literature values [20]. These compounds were identified as phenazine-1-carboxylic acid (**1**), 2-hydroxy-phenazine (**2**), and phenazine-1-carboxylic acid 2-ol (**3**) (Fig. 2D). Compounds **1**–**3** were re-dissolved in DMSO and added to the culture medium, and the callus was cultured for two weeks. Subsequently, cryptotanshinone was quantified using LC-MS/MS analysis. Compound **1** showed the most pronounced increase in cryptotanshinone production among the tested compounds (Fig. S2).

**Fig. 3:**
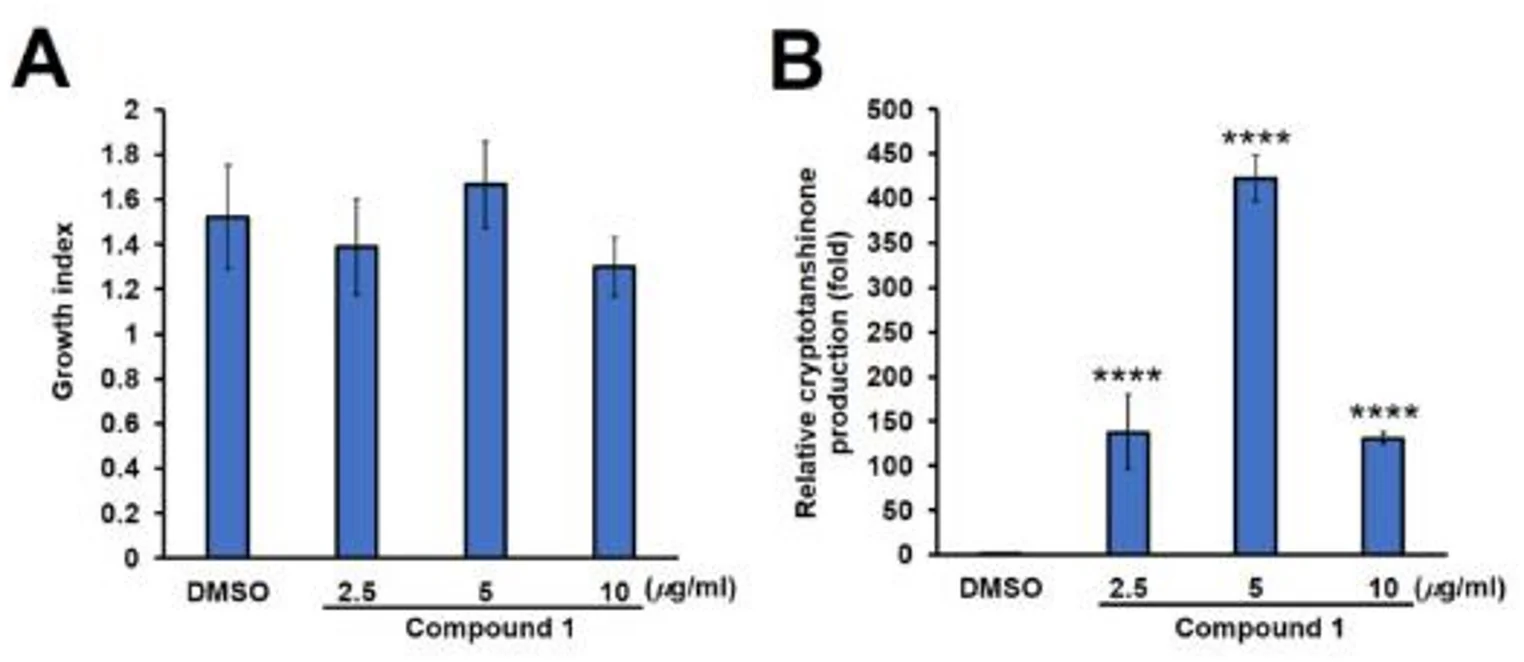
Effects of compound **1** on cryptotanshinone production in *S. miltiorrhiza* callus. **A** Growth index of callus treated with DMSO (control) or compound **1** (2.5, 5, and 10 *µ*g/ml) for two weeks; **B** Relative cryptotanshinone production in callus under the same treatment conditions, as determined by LC-MS/MS analysis. Data are presented as means ± SD, *n* = 6, *P < 0.001 versus the control culture

Based on these results, **1** was selected for further experiments. Namely, MS media supplemented with DMSO or **1** re-dissolved in DMSO (2.5, 5, and 10 µg/ml) were prepared, and callus was cultured for two weeks. Subsequently, cryptotanshinone was quantified using an LC-MS/MS system with slight modifications based on a previously reported method [17]. The results showed that the growth index was not significantly affected by treatment with **1** (Fig. 3A). In contrast, cryptotanshinone production was significantly increased at all tested concentrations, with the highest activity observed at 5 *µ*g/ml (Fig. 3B). Similar to cryptotanshinone, abietane-type diterpenes derived from cryptotanshinone, including tanshinone I, tanshinone IIA, and dihydrotanshinone [2], were also quantified using an LC-MS/MS system and a previously reported method, with slight modifications [17–19]. Increased production of these compounds was observed (Fig. S3). Compound **1** has a structure distinct from that of cryptotanshinone precursors; therefore, we deduced that **1** induce the expression of biosynthetic genes located upstream in the cryptotanshinone biosynthetic pathway. Compound **1** is a phenazine-type secondary metabolite produced by various microorganisms [21] and is widely used as a biocontrol agent because of its broad-spectrum antibacterial and antifungal activities against plant pathogens. In addition, phenazines, including **1**, have been reported to influence plant growth and induce resistance responses [22, 23]. However, the effects of **1** on the biosynthesis of plant secondary metabolites remain unclear. The results of the present study demonstrated that **1** significantly increased cryptotanshinone production without affecting cell growth, suggesting that **1** may function as an elicitor of secondary metabolism in plants.

**Fig. 4:**
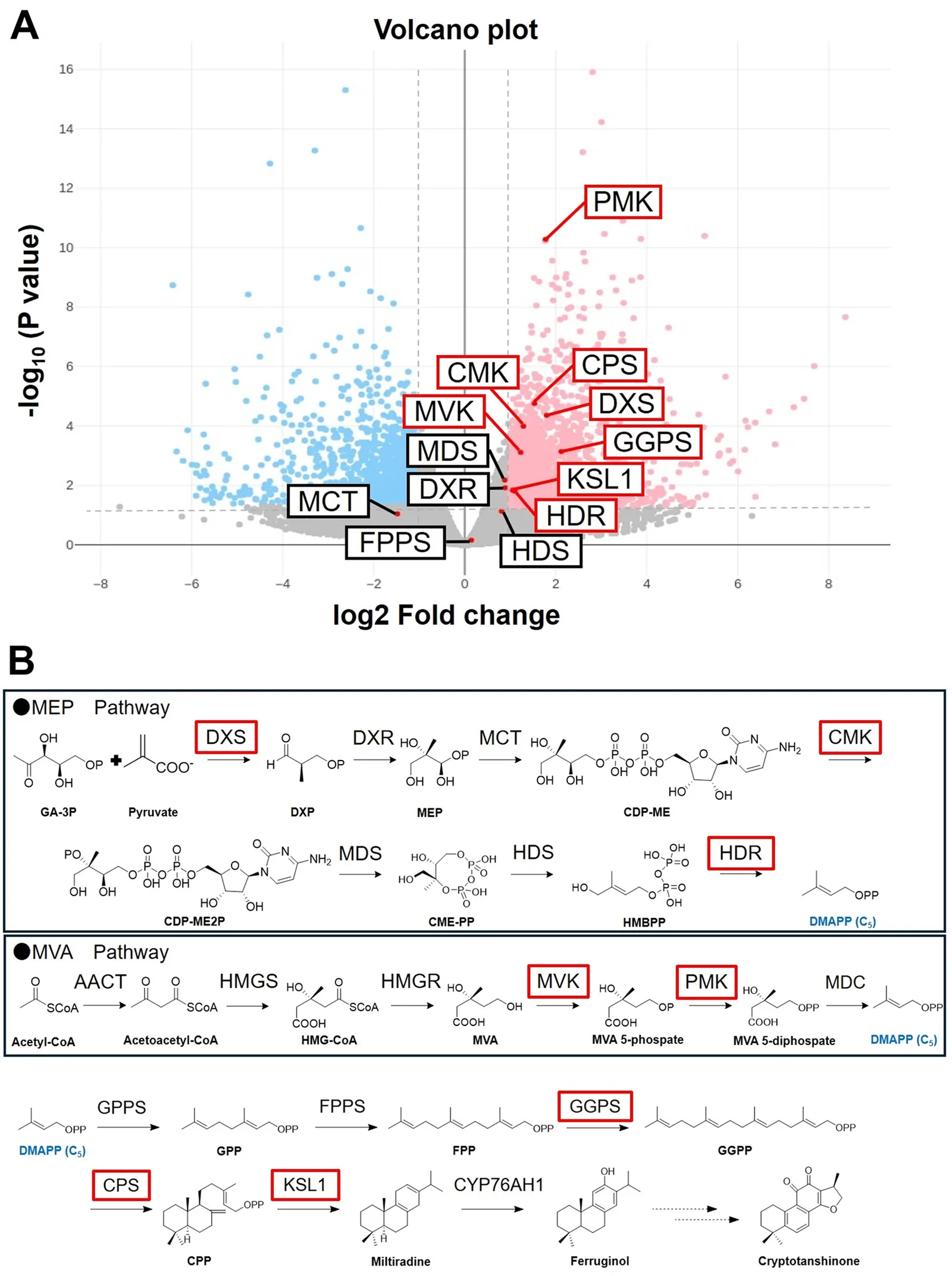
RNA-seq analysis of gene expression changes induced by **1** in *S. miltiorrhiza* callus. **A** Volcano plot showing the distribution of DEGs between **1**-treated (5 µg/ml) and DMSO-treated control samples. DEGs were defined as genes with |log2 fold change (FC)| ≥ 1 and *P* < 0.05. Red dots represent upregulated genes, blue dots represent downregulated genes, and gray dots represent genes with no significant changes. Representative genes involved in tanshinone biosynthesis are highlighted; **B** Schematic representation of the tanshinone biosynthetic pathway, including the mevalonate (MVA) and methylerythritol phosphate (MEP) pathways and downstream diterpenoid biosynthesis. Genes highlighted in red indicate those that were significantly upregulated in response to treatment with **1**

To elucidate the effect of **1** on the expression of tanshinone biosynthesis-related genes in *S. miltiorrhiza* callus, RNA-seq analysis was performed using callus samples treated with **1** redissolved in DMSO (5 µg/ml) and DMSO-treated controls under identical culture conditions. Differentially expressed genes (DEGs) were screened using the criteria of |log2 fold change (FC)| ≥ 1 and *P* < 0.05. A result, a total of 3,206 DEGs were identified, and visualized using a volcano plot (Fig. 4A, Table S1). Volcano plot analysis revealed that numerous genes were significantly upregulated or downregulated, indicating that **1** induces widespread changes in gene expression. In particular, a set of genes involved in tanshinone biosynthesis was distributed in the upregulated region of the plot. Tanshinone biosynthesis proceeds via the mevalonate (MVA) and methylerythritol phosphate (MEP) pathways, which provide isoprenoid precursors for downstream diterpenoid biosynthesis [2]. Further analysis of this biosynthetic pathway showed that **1** upregulated the expression of multiple genes encoding key enzymes involved in tanshinone biosynthesis, as illustrated in Fig. 4B. These results indicate that **1** increases cryptotanshinone production by inducing the expression of genes involved in its biosynthesis in *S. miltiorrhiza* callus.

In the present study, we evaluate the amount of tanshinones with two weeks incubation after stimulation by bacteria extracts or **1**–**3**. Because we discussed two weeks are enough time to produce tanshinones after stimulation. In a previous study, the expression levels of most genes in the MEP pathway were increased at the 36 h time point after stimulation by the carbohydrate fraction of yeast extract with Ag^+^ in hairy root. And the amount of many tanshinone-related compounds began to increase at 48 h and exhibited dramatic increases at 120 h and 240 h [24]. Therefore, it is necessary to evaluate the time-dependency of tanshinones production after stimulation by **1**.

**Fig. 5:**
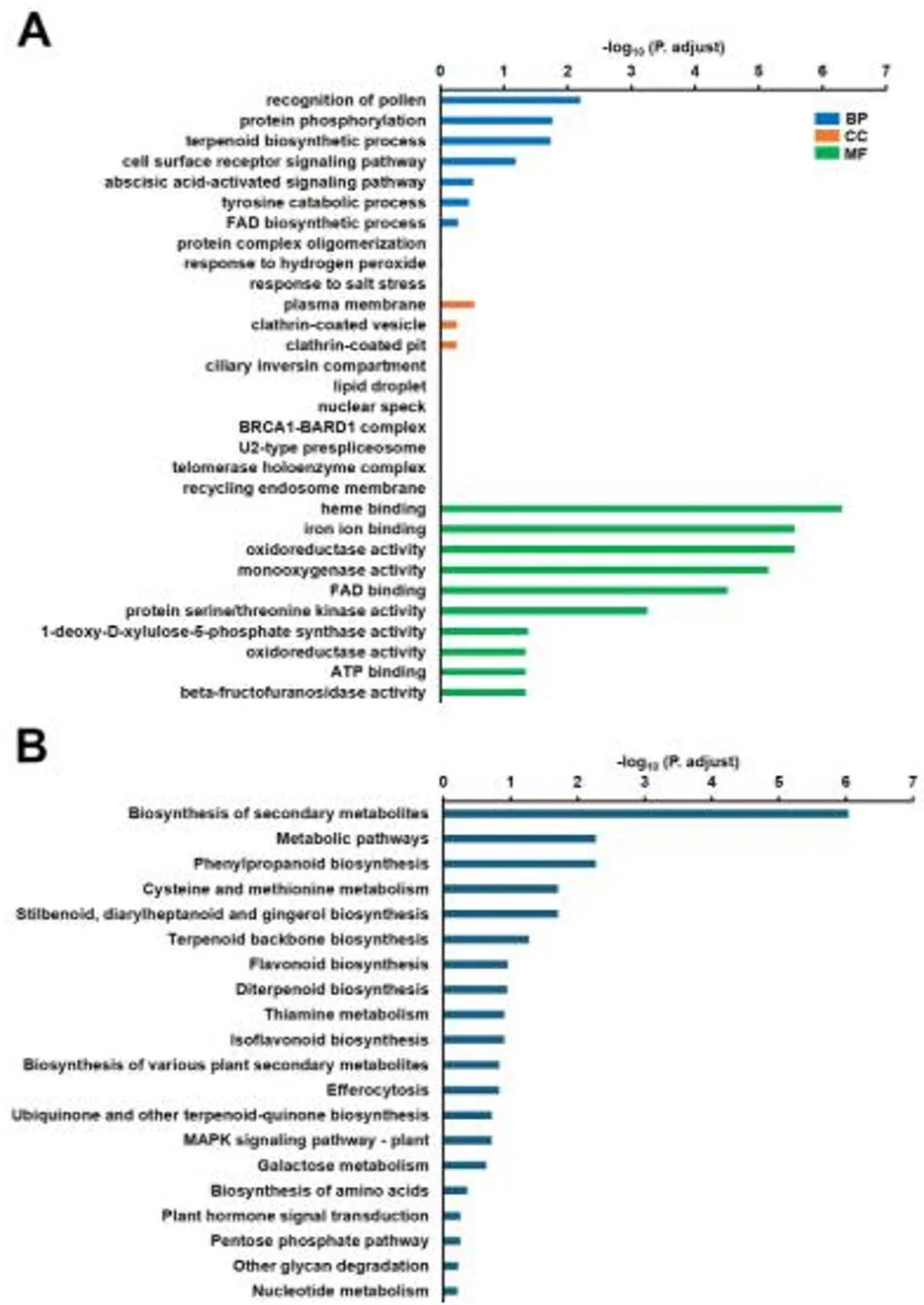
Functional enrichment analysis of DEGs in response to treatment with **1** in *S. miltiorrhiza* callus. **A** GO enrichment analysis of DEGs. The enriched GO terms are classified into three functional categories: biological process (BP), cellular component (CC), and molecular function (MF). The x-axis indicates the GO terms, and the y-axis represents the enrichment significance expressed as −log10 (adjusted *P*-value); **B** KEGG pathway enrichment analysis of DEGs. The top 20 significantly enriched pathways in the group treated with **1** are shown. The x-axis represents the enrichment significance {−log_10_ (adjusted *P*-value)}

To gain further insight into the transcriptional changes induced by **1**, Gene Ontology (GO) enrichment analysis was performed. DEGs were mainly classified into three functional categories: biological process (BP), cellular component (CC), and molecular function (MF). The GO terms with the highest numbers of DEGs in the BP, CC, and MF categories were GO: 0048544 (recognition of pollen, 11 DEGs), GO: 0005886 (plasma membrane, 52 DEGs), and GO: 0020037 (heme binding, 35 DEGs), respectively (Fig. 5A, Table S2). Among the three GO categories, MF showed the highest enrichment significance, whereas BP and CC showed a relatively low significance. In particular, MF terms, such as oxidoreductase activity, heme binding, monooxygenase activity, and kinase activity were prominently enriched, suggesting that **1** mainly affects enzymatic and catalytic functions at the molecular level. The identified DEGs were mapped to the Kyoto Encyclopedia of Genes and Genomes (KEGG) database to investigate the biological pathways involving these genes. Subsequently, KEGG enrichment analysis was performed, and the top 20 significantly enriched pathways in the group treated with **1** are shown in Fig. 5B (Table S3). KEGG enrichment analysis revealed that pathways related to secondary metabolism were predominantly enriched in the group treated with **1**. Pathways such as terpenoid backbone, diterpenoid, phenylpropanoid, and flavonoid biosynthesis were significantly enriched. These results suggest that **1** enhances secondary metabolite production by activating both precursor supply pathways and downstream biosynthetic processes.

Cryptotanshinone has been identified as a phytoalexin in *S. miltiorrhiza* and is involved in the passive and active plant defense response to pathogens [25]. In this study, **1** produced by *P. chlororaphis* KPUPH-SMC promoted tanshinones production. These results suggest that *P. chlororaphis* KPUPH-SMC may activate the defense response of its host plant through **1**, which may contribute to the establishment of a balanced mutualistic interaction between the bacterium and the host plant. Although the effects of **1** were evaluated in callus cultures, the results may not fully represent those in differentiated tissues. Therefore, further studies using whole plants are required to evaluate its effects on plant growth, secondary metabolite accumulation, and plant-endophyte interactions.

## Conclusions

In this study, compound **1**, produced by the endophyte *Pseudomonas chlororaphis* KPUPH-SMC, was demonstrated to act as an effective elicitor that increased tanshinone biosynthesis in *S. miltiorrhiza* callus without inhibiting cell growth. Transcriptome and enrichment analyses revealed that **1** upregulated genes involved in tanshinone biosynthesis. Moreover, these results suggest that secondary metabolites produced by endophytes play an important role in plant–endophyte interactions by modulating host metabolic pathways. Such interactions may contribute to the regulation of secondary metabolism in medicinal plants and may be useful for improving the quality and production efficiency of medicinal compounds.

## Experimental

### General

EIMS and HREIMS data were collected using a JEOL JMS-GCMATE mass spectrometer. ^1^H NMR spectroscopy was performed using JEOL ECS400 (400 MHz) and JNM-ECA 600 (600 MHz) spectrometers. ^13^C NMR spectroscopy was performed using a JNM-ECA 600 spectrometer (150 MHz).

Reversed-phase silica gel column chromatography was performed using a C_18_-OPN column (Nacalai Tesque Co., Inc., 140 μm). Reversed-phase high-performance TLC was performed using TLC plates pre-coated with RP-18 WF_254S_ silica gel (Merck, 0.25 mm). HPLC was performed using a Shimadzu SPD-M20 UV-vis detector. YMC-Triart C18 (250 × 4.6 mm i.d.) columns were used for analytical and preparative purposes.

### Plant material

Apical buds and roots were collected from *Salvia miltiorrhiza* grown at the Medical Botanical Garden of Kyoto Pharmaceutical University in July 2024. The plant materials were identified by one of the authors (J. T.). A voucher specimen of the medicinal plant (KPU-2406) was deposited in the garden.

### Callus induction and culture

MS medium [25] containing vitamins was used for all callus experiments. The MS medium was supplemented with 30 g/l sucrose, 0.5 ml/l Plant Preservative Mixture™ (PPM™), 2,4-dichlorophenoxy acetic acid, 0.5 mg/l kinetin, and 3 g/l gellan gum and was autoclaved at 121 °C for 20 min [26].

The explants were soaked in 70% ethanol for 1 min, followed by 5% sodium hypochlorite for 5 min. Then, the explants were immersed in a 5% PPM™ solution and sterilized, after which they were rinsed with distilled water three times. Sterilized explants were inoculated on the media and cultured under dark conditions in a growth chamber at 25 ± 2 °C for approximately four weeks. After callus formation was confirmed, the successfully induced callus was isolated from the explant, transferred to the medium, and cultured under dark conditions in a growth chamber at 25 ± 2 °C. Calluses were passed on at approximately two-week intervals.

### Isolation of bacterial endophytes

*S. miltiorrhiza* endophytic bacteria were isolated using the surface sterilization method described by Li et al. [27]. Briefly, roots were washed with water and soaked in 75% ethanol for 1 min. Then, samples were soaked in 5% sodium hypochlorite for 3 min, followed by three washes with sterile distilled water. The surface-sterilized samples were cut into 0.5 cm × 0.5 cm segments using sterile tweezers. The segments were inoculated with potato dextrose agar (PDA) and 0.1 g/l chloramphenicol and incubated at 28 °C for three days. After incubation, each bacterial colony that emerged was picked up and repeatedly streaked on PDA to produce single colonies.

### Identification of the KPUPH-SMC strain

The DNA of the endophytic bacteria in *S. miltiorrhiza* was extracted according to the method described by Makimura et al. [28]. The DNA was subsequently used as a template for polymerase chain reaction (PCR) amplification with the universal primers 27 F (5′-AGAGTTTGATCCTGGCTCAG-3′) and 1492R (5′-GGTTACCTTGTTA CGACTT-3′). The amplified 16 S rRNA gene region (507 bp; GenBank accession number PZ626786) of strain KPUPH-SMC was obtained, and BLAST analysis using the National Center for Biotechnology Information (NCBI) database revealed 100.00% sequence identity with *Pseudomonas chlororaphis* (accession number CP118151.1).

### Cultivation of KPUPH-SMC and extraction of metabolites

The seed culture of KPUPH-SMC was prepared in 90 mm Petri dishes containing PDA medium at 28 °C for seven days. The seed culture was cut into small pieces and added to 120 90 mm Petri dishes containing PDA medium. The cultures were milled by liquidizer (MJA-G100, Yamazen, Osaka) after incubation at 28 °C for two weeks. Then, H_2_O was added to the milled cultures, followed by partitioning with EtOAc (1.06 g). A portion of the EtOAc extract (190 mg) was re-dissolved in DMSO for biological assays, while the remaining extract (870 mg) was fractionated using reversed-phase column chromatography.

### Effect of *P. chlororaphis* KPUPH-SMC on tanshinones production in *S. miltiorrhiza*

The KPUPH-SMC EtOAc-soluble fraction was dissolved in DMSO. Calluses obtained two weeks after subculture (1 g fresh weight) were inoculated on to MS medium (30 ml) containing the EtOAc soluble fraction (25 and 50 *µ*g/ml). Calluses grown on the same MS medium with only DMSO were used as the control group. The cultures were placed at 25 ± 2 °C in a growth room. After two weeks of growth, the calluses were harvested and their fresh weights were measured. The growth index was calculated as follows: Growth index = callus fresh weight after two weeks of culture / callus fresh weight before cultivation. The calluses were freeze-dried in a lyophilizer (EYELA FDU-1200). The lyophilized calluses were crushed and extracted using isopropyl alcohol (1 ml) with sonication for 15 min at 37 °C. The extracts were centrifuged at 2500 rpm for 5 min. The extraction process was repeated twice. The solution was filtered through a syringe filter (0.45 *µ*m) and subjected to LC-MS/MS analysis.

LC-MS/MS analysis of tanshinones was performed using an LC-MS/MS system as reported previously, with modifications [17–19]. The LC-MS/MS system consisted of an HPLC system (Shimazu Corporation, Kyoto, Japan) coupled to a triple quadrupole mass spectrometer with an electrospray ionization interface. Chromatographic separation was performed using an YMC-Triart C18 column (3 *µ*m, 150 × 2.0 mm i.d.) maintained at 30 °C. The flow rate was 0.2 ml/min, and the injection volume was 50 *µ*l. The mobile phase consisted of 0.3% CH₃COOH in H_2_O as solvent A and CH_3_CN as solvent B. The column was eluted under isocratic conditions with 60% solvent B for 30 min. The operating parameters for MS detection were as follows: nebulizer gas flow, 3 l/min; drying gas flow, 15 l/min; desolvation line temperature, 200 °C, heat block temperature, 400 °C; and interface voltage; 4.5 kV. Quantification was performed using multiple reaction monitoring (MRM) at transitions of *m/z* 295.10→277.25 for tanshinone IIA [17], *m/z* 297.00→251.25 for cryptotanshinone [17], *m/z* 277.20→178.10 for tanshinone I [18], and *m/z* 279.00→261.15 for dihydrotanshinone [19]. Data acquisition and quantitative analysis were performed using LabSolutions software (Shimazu, Kyoto, Japan). A mixed working solution for calibration containing tanshinone I, tanshinone IIA, cryptotanshinone and dihydrotanshinone was prepared by dilution with methanol and subsequently diluted with methanol to yield calibration standards at 0.01, 0.1, 1, 10, and 100 ng/ml. The concentrations of the tanshinones were determined by plotting the peak area ratios of the analytes to the calibration standards. The mean and standard deviation (SD) were calculated using Microsoft Excel.

The KPUPH-SMC EtOAc soluble fraction (870 mg) was separated by reversed-phase silica gel column chromatography [MeOH–H_2_O (1:9→2:8→3:7→5:5→7:3→9:1, v/v) → MeOH] to give four fractions [Fr. 1 (45 mg), Fr. 2 (50 mg), Fr. 3 (433 mg), and Fr. 4 (118 mg)]. Each fraction was dissolved in DMSO (10 *µ*g/ml) and added to the culture medium. Cultivation, extraction, and LC-MS/MS analysis were performed under the same conditions as described above. The active fraction (Fr. 2) was purified using HPLC [mobile phase: CH_3_CN–0.3% CH_3_COOH in H_2_O (40:60, v/v), flow rate: 5 ml/min, column: YMC Triart C-18 (5 *µ*m, 250 × 10 mm i.d.), and λ: 254 nm] to give **1**. The isolated compounds were identified by comparing their physical data (^1^H-NMR and, ^13^C-NMR) with reported values. Compound **1** was dissolved in DMSO (2.5, 5, and 10 *µ*g/ml) and added to the culture medium. Cultivation, extraction, and LC-MS/MS analysis were performed under the same conditions as described above.

### Statistical analysis

Statistical analysis was performed using GraphPad Prism 8.21 software. Statistical analysis was performed using a one-way analysis of variance (ANOVA), followed by Dunnett’s test. Differences were considered significant at *P < 0.001. 

### Extraction of total RNA and cDNA library construction

The calluses of the treated and control samples were collected for mRNA sequencing with three biological replicates. Total RNA was extracted using the Maxwell RSC Plant RNA Kit according to the manufacturer’s instructions. The RNA concentration and purity were measured using a NanoDrop One (Thermo Fisher Scientific) and TapeStation 4150 (Agilent Technologies). Sequencing libraries were generated using a NEBNext Poly (A) mRNA Magnetic Isolation Module and NEBNext Ultra II Directional RNA Library Prep Kit following the manufacturer’s recommendations.

### Transcriptome sequencing and DEG enrichment analysis

Library preparations were sequenced on an Illumina NovaSeq X Plus platform with a sequencing read length of 150 bp (PE150). The raw data were processed using Trimmomatic software (version 0.38), and the clean data were obtained by trimming low-quality (< 20) bases and removing reads containing adapter sequences with the following parameter: ILLUMINACLIP. These clean reads were then mapped to the reference genome sequence of *S. miltiorrhiza* using RNA-seq aligner HISAT2 (Version 2.1.0). Gene expression levels were normalized to transcripts per million (TPM), and differential expression analysis between the treated and control groups was performed using the DESeq2 package (version 1. 24. 0) with relative log expression (RLE) normalization. DEGs were identified using thresholds of |log2 fold change| > 1 and an adjusted *P* value < 0.05 based on the Benjamini–Hochberg (BH) method. GO enrichment analysis of DEGs was performed using GOATOOLS (version 1.1.6), and KEGG enrichment analysis was conducted using the clusterProfiler R package (version 4.10.0) after annotation with the KEGG database. *P* values < 0.05 were considered to indicate significant enrichment.

## Supplementary Information

Below is the link to the electronic supplementary material.

LC-MS/MS analysis of tanshinones in *Pseudomonas chlororaphis* KPU-SMC extract, effects of **1**–**3** on cryptotanshinone production in *S. miltiorrhiza* callus, effects of **1** on tanshinones (tanshinone I, tanshinone IIA, and dihydrotanshinone) production in *S. miltiorrhiza* callus (Supplementary file 1, Word file)

The list of DEGs used for volcano plot analysis (Supplementary file 2, Excel file)

The list of enriched GO terms in the **1**-treated group (Supplementary file 3, Excel file)

KEGG pathway enrichment of upregulated genes in the **1**-treated *S. miltiorrhiza* callus transcriptome (Supplementary file 4, Excel file) are available free of charge via the Internet 1
