# Supplementary material for: The endophyte secondary metabolite phenazine-1-carboxylic acid promotes tanshinone biosynthesis in Salvia miltiorrhiza callus
Source: J Nat Med. 2026 Oct 5;80(6):1662–71. doi: 10.1007/s11418-026-02075-w (PMC13723477; doi:10.1007/s11418-026-02075-w)
Supplement: Supplementary file 1 [file 11418_2026_2075_MOESM4_ESM.docx]

The endophyte secondary metabolite phenazine-1-carboxylic acid promotes tanshinone biosynthesis in *Salvia miltiorrhiza* callus

Miho Hamai^1^ • Anju Ishii^1^ • Nozomi Sakamoto^1^ • Hiroki Fujioka^1^• Junko Tsukioka^1^• Tomoe Ohta^1^ • Seikou Nakamura^1^ • Takahiro Matsumoto^1*^

1. Kyoto Pharmaceutical University, Misasagi, Yamashina-ku, Kyoto 607-8412, Japan.

**List of Supporting Information**

Fig. S1	LC-MS/MS analysis of tanshinones in Pseudomonas chlororaphis KPU-SMC extract.

Fig. S2	Effects of **1**–**3** on cryptotanshinone production in S. miltiorrhiza callus.

Fig. S3	Effects of **1** on tanshinone I, tanshinone IIA, and dihydrotanshinone productions in S. miltiorrhiza callus.

**Fig. S1** LC-MS/MS analysis of tanshinones in Pseudomonas chlororaphis KPU-SMC extract.

LC-MS/MS SRM chromatograms dihydrotanshinone, cryptotanshinone, tanshinone I, and tanshinone IIA in Pseudomonas chlororaphis KPU-SMC extract. Column, YMC-Triart C18 column (5 μm, 250×4.6 mm i.d.); mobile phase, CH_3_CN–0.3% CH_3_COOH in H_2_O (6:4); flow rate: 1.0 ml/min.

**Fig. S2** Effects of **1**–**3** on cryptotanshinone production in S. miltiorrhiza callus

Relative cryptotanshinone production in callus treated with DMSO (control) or compound 1–3 (5 *μ*g/ml) for two weeks, as determined by LC-MS/MS analysis. Data are presented as means ± SD, *n* = 5, **P* < 0.001 versus the control culture.

**Fig. S2** Effects of **1** on tanshinone I, tanshinone IIA, and dihydrotanshinone productions in S. miltiorrhiza callus

Relative tanshinone I, tanshinone IIA, and dihydrotanshinone productions in callus treated with DMSO (control) or compound 1 (2.5, 5, and 10 *μ*g/ml) for two weeks, as determined by LC-MS/MS analysis. Data are presented as means ± SD, *n* = 6, **P* < 0.001 versus the control culture.
